# Five lessons from a mid-level health manager intervention to increase uptake of tuberculosis prevention therapy in Uganda: ‘it is a completely different thing to implement what you know.’

**DOI:** 10.1080/16549716.2024.2427434

**Published:** 2024-11-18

**Authors:** Jason Johnson-Peretz, Canice Christian, Cecilia Akatukwasa, Fred Atwine, Elijah Kakande, Moses R. Kamya, Diane V. Havlir, Carol S. Camlin, Gabriel Chamie

**Affiliations:** a Department of Obstetrics, Gynecology, & Reproductive Sciences, University of California, Oakland, CA, USA; b Department of Epidemiology and Biostatistics, University of California, San Francisco, CA, USA; c Infectious Diseases Research Collaboration (IDRC), Kampala, Uganda; d Department of Medicine, Makerere University College of Health Sciences, Kampala, Uganda; e Department of Medicine, University of California, San Francisco (UCSF), Center for AIDS Prevention Studies, San Francisco, CA, USA; f Department of Medicine, Division of HIV, Infectious Diseases & Global Medicine, University of California, San Francisco, CA, USA

**Keywords:** Decision space, healthcare, capacity building, decentralization, tuberculosis, HIV, management, implementation

## Abstract

**Background:**

Leadership skills are essential for middle-level healthcare manager efficacy. Capacity-building efforts may attempt behavioural change by filling ‘knowledge gaps’ while neglecting a sustainable application of that knowledge. Sustainable application of that knowledge, or implementation know-how, must resonate with local cultural patterns. When it is neglected, root issues like unclear decision-making space and local authority to interpret policy during implementation remain unaddressed. Particularly in decentralized healthcare systems, the impact can appear in implementation challenges, subjective decision-making, poor teamwork, and an absence of disseminating best practices.

**Objectives:**

The SEARCH-IPT trial led a series of mini-collaborative meetings, which provided business leadership and management training for an intervention group of mid-level healthcare system managers in rural Eastern, East-Central, and Southwestern Uganda to see whether this would increase uptake of isoniazid-prevention therapy (IPT) for people living with HIV (PLHIV) in intervention districts. IPT is known to reduce active tuberculosis (TB), a leading cause of death among PLHIV, by 40–60%.

**Methods:**

We performed a thematic analysis of six focus-group discussions from this intervention (held in May 2019, January 2020, September 2021) and 23 key informant interviews with control group participants (between February and August 2019 and September and December 2020).

**Results:**

Analysis revealed five implementation skill sets District Health Officers (DHOs) and District Tuberculosis and Leprosy Supervisors (DTLSs) deployed to achieve sustainable implementation and realize their decision-making space. The five practices were as follows: data-based decision-making, root-cause analysis, quality assurance, evidence-based empowerment, and sharing best practices with colleagues.

**Conclusion:**

These practices reached beyond outcome measures to address root problems around the DHO’s range of authority and elicit buy-in from district health workers. For successful capacity building at the mid-manager level, focusing on core practices as part of competency is objectively implementable and measurable at the system level and does not rely on DHO self-assessments.

## Paper context

- **Main findings:** District Health Officers and District Tuberculosis and Leprosy Supervisors deployed five implementation skill sets to achieve sustainable implementation and clarify ambiguous decision-making space: data-based decision-making, root-cause analysis, quality assurance, evidence-based empowerment, and sharing best practices with colleagues, thereby reaching beyond outcome measures to address root problems around the District Health Officer’s range of authority and obtain buy-in from district health workers.
- **Added knowledge**: Horizontal accountability, in which mid-level health system managers share their experiences implementing core practices, has made concrete and demonstrably sustainable implementation changes at the district level, suggesting that capacity building at the mid-manager level must reach further than identifying knowledge gaps and instead additionally show people how to implement knowledge they may already possess.
- **Global health impact for policy and action**: Focusing on core practices – rather than competencies – is objectively implementable and measurable at the system level and does not rely on self-assessments of knowledge or pre/post-training skill sets that have been shown to be unreliable in predicting the sustainability of training lessons.

## Background

Leadership involves galvanizing and guiding change; management concerns itself with performance oversight [1]. Leadership development is an upstream intervention in health systems, mediated by downstream system factors like staff motivation and resource allocation that require management and performance oversight before directly impacting patients [1]. Both leadership and management skills are fundamental for mid-level manager efficacy in decentralized healthcare systems. In decentralized contexts, mid-level managers like Uganda’s District Health Officers (DHOs) sit at the intersection of overseeing the implementation of Ministry of Health (MoH) directives while directing locally deployed, community-tailored programmes [2]. However, unlike in the private sector, many mid-level healthcare managers do not attend business schools or take courses to develop these skills [3].

For DHOs to effectively wield the authority transferred to them from the MoH, they must have both management capacities to oversee staff implementation and adequate decision-making space to lead effectively [4]. Although leadership development is one component of capacity building at the middle-management level, efforts sometimes attempt to effect behavioural change by filling ‘knowledge gaps’ to foster management efficacy and promote leadership – while neglecting implementation know-how as a central component in core competency training [5,6]. By ‘implementation know-how’, we mean the sustainable application of core practices in localized leadership contexts where sharing effective practices enhances competencies. In certain decentralized contexts, unclear boundaries around ‘decision-making space’ – the degree to which mid-level managers, as local authorities, are empowered by official policy to interpret policy locally or make decisions for that locale – further complicate the situation [2,7–10]. The impact of limited skills training and unclear system-level range of authority for mid-level managers can lead to implementation challenges, non-evidence-based decision-making, poor teamwork, and limited dissemination of best practices [8,11].

These capacity gaps have detrimental effects on health outcomes for people living with HIV (PLHIV). For example, the WHO has recommended Isoniazid preventative therapy (IPT) to reduce the risk of active tuberculosis in PLHIV [12,13]. Despite this recommendation, IPT uptake remains low across sub-Saharan Africa [14]. In Uganda, a high TB burden country, less than 2% of PLHIV had received IPT by 2018. Yet in contrast to other countries, Uganda has otherwise been an exceptionally successful case of decentralisation, so what might explain this lack of uptake [15]? Because of Uganda’s decentralized healthcare structure, district health leaders are responsible for implementing IPT within the country. That decentralization, however, means that each district leader is left to his or her own devices and learning curve, which can vary depending on the leader’s existing social capital or network strength [4,16]. Studies have shown that Uganda has a fairly collectivist culture, with a short-term orientation and high uncertainty avoidance, both of which influence managerial styles and conflict resolution [17,18]. Examining what culture-specific leadership and management styles make the decentralisation process more or less successful is thus an open area of inquiry, in Uganda and Africa more generally [1].

Efforts to tailor interventions to specific cultural or community contexts have been aided by the two-part PRECEDE–PROCEED model [19,20]. In the first portion (PRECEDE), policymakers establish community-prompted, desired results; they then set priorities for removing obstacles or attaining the goal, identify predisposing factors that affect those priorities, and examine the administrative or policy constraints which influence what can be implemented. The PROCEED portion of the model then encompasses implementation and subsequent intervention evaluations around process, impact, and outcome. How such interventions can be sustained within a given cultural or geographic context by reporting identifiable and contextually specific implementation skills after the intervention ends, however, is less often reported.

The NIH-funded SEARCH-IPT trial, taking a cue from the PRECEDE model, identified three barriers to IPT uptake at the district level: lack of knowledge about the efficacy of IPT, a system already operating at capacity due to existing financial and personnel constraints, and diluted managerial efficacy due to poor infrastructure. To address these challenges, SEARCH-IPT led a series of mini-collaborative meetings, which provided business leadership and management training for an intervention group of mid-level healthcare system managers in rural Eastern, East-Central, and Southwestern Uganda. The intervention sought to increase needed collaboration between districts through skills training and capacity building within the decentralized healthcare context to address disease spillover between districts and increase uptake of IPT for tuberculosis among PLHIV.

Notably, the SEARCH intervention overlapped with a national, centralized push to scale up IPT. Quantitative results demonstrated that the intervention districts evinced greater ‘staying power’ after that push ended, sustaining high levels of IPT initiation compared to both control districts and other districts in the country – not only after the national push ended but also during the COVID-19 pandemic [21]. These results suggest the intervention addressed certain public health challenges that had previously remained intractable.

This paper reports identifiable, contextually specific implementation skills that appear to have promoted that sustainability. We suggest that the mechanism behind the intervention’s sustainability lies in five core skills the intervention participants identified as most useful for teaching the know-how of implementation, and highlight their relation to two addressable root issues mid-level health managers may face in a decentralized national healthcare context.

## Methods

### Context

In 2017 and 2018, the SEARCH-IPT trial was deployed across 82 rural Ugandan health districts (out of 135 districts total) in preparation for the intervention period (2019–2021). Cluster-randomisation allocated 39 to the control arm and 43 to the intervention. In the intervention arm, we convened groups to disseminate knowledge about IPT, and we provided comparative data to the DHOs on IPT uptake in their and neighbouring intervention districts. The primary outcome was increased IPT initiation for adults with HIV and integration of IPT as standard of care. We included *qualitative* interviews as part of the process, impact, and outcome evaluations. A detailed description of the *quantitative* methods and primary statistical outcomes has been published elsewhere; briefly, while both arms showed increased initiation of IPT during a Ministry of Health-led national campaign, the intervention arms maintained their levels of initiation one year after the campaign ended, while control arm districts did not [21].

### Qualitative data

The qualitative portion of the study employed six focus group discussions (FGDs) following a semi-structured question format among intervention-site DHOs and DTLS participants over the three-year study period (May 2019, January 2020, September 2021). Following each meeting, we invited all mini-collaborative participants to participate in the FGDs (lasting an average of 111 minutes). As described elsewhere, the number of participants at each FGD varied between 7 and 11 people; all but two participants were male, reflecting the overall gender distribution among health district leadership in Uganda [21–23]. Most participants were middle-aged and had held their positions for more than two years. The FGD guides prompted discussion around participant perspectives on the intervention content and the changes it effected. Additionally, we asked participants about changes in communication styles, resource management, relations with policymakers and stakeholder engagement more generally. A team of trained qualitative researchers, CA, FA, RB, HI, moderated the FGDs and participated in the data collection activities.

We paired the FGD data with 23 in-depth, semi-structured key informant interviews (KIIs) with control group DHO or DTLS participants, conducted in private and convenient locations, between February and August 2019 and September and December 2020. Each lasted an average of 37 minutes; all participants were male. Like the FGDs, only one KIIs participant was in his 30s, and only one had held their position for less than two years; the rest were more mature in both age and experience in their role. FGD participants received transportation reimbursement, while researchers travelled to the KII sites. All participants provided written informed consent. Both FGDs and KIIs were recorded, transcribed, and then analyzed.

### Analysis

**Figure 1.**
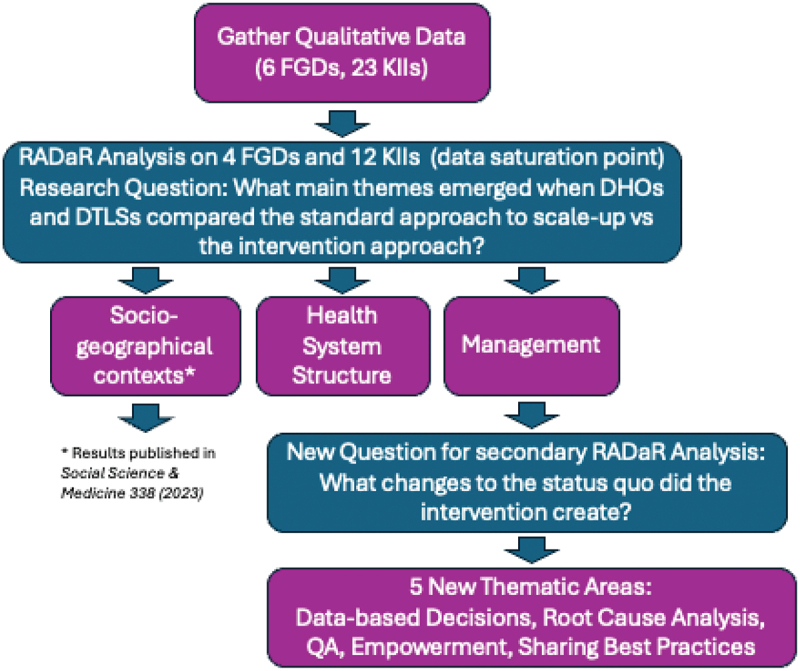
Qualitative analysis process. Acronyms are as follows – FGD: focus group discussion; KII: key informant interview; DHO: District Health Officer; DTLS: District Tuberculosis and Leprosy Supervisor; QA: quality assurance.

The first author and primary qualitative analyst for this paper, JJP, was trained in qualitative methods via an MPhil degree in Medical Anthropology (Oxon). Blinded to both the statistical outcomes and the language used in the training, he used a Rigorous and Accelerated Data Reduction (RADaR) technique, a variant of framework analysis, to reduce data and identify main themes [24]. (Figure 1) The main research question focused on perspectives towards top-down approaches to IPT scale-up compared to intervention methods, and self-reflection on changes in district management’s leadership skills. Although we conducted a total of 6 FGDs and 23 KIIs, we reached data saturation after an initial RADaR analysis of the first 4 FGDs and 12 KIIs representing each region, which we confirmed by reading the remaining FGD and KII transcripts.

The first author uncovered preliminary thematic groups divisible into management, health system structure, and socio-geographical context [22]. To identify what the intervention may have inadvertently engendered, further reduction of the ‘management’ thematic group focused specifically on changes to the status quo introduced by the intervention training. Still blinded, JJP then organized these themes around five main practices the intervention districts implemented and that were mostly absent in the control group interviews. Within each section, the authors have subsequently attempted to retain reports of both challenges and, especially from intervention participants, solutions to those challenges.

## Results

We present the results by first describing the five core intervention practices that our participants repeatedly returned to as addressing what turned out to be two underlying concerns, which we present second. As mentioned in the Methods section, these core practices do not necessarily coincide with the training materials; rather, they were derived from inductive thematic analysis. The five core practices were as follows: data-based decision-making, root-cause analysis, quality assurance, evidence-based empowerment, and sharing best practices with colleagues. We contrast the results from the intervention groups with observations offered by control group participants to highlight how those five core practices reached beyond outcome measures to suggest two root problems – a lack of clarity around the DHO’s range of authority (a leadership issue) and challenges in getting buy-in from the district health workers (a management challenge) – that consistently confronted our participants, suggesting why the intervention participants valued those five practices. In the discussion, we elaborate how the core practices and root issues intersect in light of emerging challenges in decentralized healthcare settings.

### Data-based decision-making: addressing high uncertainty avoidance through tacit consensus and horizontal accountability

During the trial, SEARCH-IPT held regular meetings where a range of data on IPT uptake was shared with the teams. Using that data became central to decision-making in the intervention arm. Objective decision-making based on data clarified the decision-making space, while sharing it gave a sense of collective action to those who implemented decisions based on that data:

For me as a manager, … I found <the data and progress report> to be a handy tool, to realign my workforce and to keep telling them that this is the target, and that we are way below; or I give them a pat on the shoulder that, “you see, we are now at 95%, keep it there because that is what we want to be.” You see, having data is very important and when you use it, you become a very objective manager. You cease leading impulsively or from a sentimental approach, you are more objective. *DHO Intervention, Southwest*

While DHOs did meet outside SEARCH in somewhat didactic, top-down meetings concerned with MoH-directed priorities, one intervention DHO noted that ‘before the start of this project, data on IPT was very negligible; that is why the health workers seemed not to focus so much on the part of IPT … ’ (*DHO Intervention, East*). Generally, outside of SEARCH-IPT, the source of IPT-specific data was gained either from Implementation Partners (IPs), which varied by district, or more commonly, from district league tables accessible to all districts. As one DTLS aptly put it, ‘When you perform well and motivated you will look at doing work better. ’ (*DTLS Control, East*)

Thus, while having comparative data motivated managers, when the manager’s ability to make objective, rather than apparently subjective decisions, became visible, a sense of ownership and recognition of rationale were returned by the team and led to managers’ greater self-confidence in their management style. Visible data-based decision-making seemed to be the most essential of the five practices in building solidarity and project buy-in among the district teams and is interrelated with the next two core practices we detail.

### Root-cause analysis through network segmentation led the DHOs to greater engagement with the full team, activating culturally valued collectivity

Managers used the IPT uptake rankings to identify the potential sources of poor or improved performance through network segmentation of the data [25–27]. Upon seeing how his district was doing, one intervention DTLS said, ‘When you see you are performing badly, you become more vigilant to sort of analyse the root cause of the problem, and you try to do something in order to improve.’ (*DTLS Intervention, East*) Another DHO specifically named segments within the care flow that might need to be addressed after he saw his dashboard standing decline:

It keeps us on our toes. Every time you look at the dashboard, you are like, “eeh, now I am declining, I am going to 60; I am going to 50; what has gone wrong? Is it the data reporting, is it the recruitment of new clients on INH or is it the completion?” So, you keep looking out for those things that are making you perform poorly. *DHO Intervention Southwest*

For this to work, two parameters in a data set are needed: comparative with other teams, and full, local accessibility. Discussing these elements with local teams helped develop a collective sense of engagement, as reports understood how that data helped the manager make better decisions:

On my side, the presentation was an eye opener because the use of data has been missing in our planning meetings, and this data opened up my mind and helped me to engage the team members so that we could discuss and see where the problem is arising from. It is like doing a quality improvement project, so it helps you to identify the gaps, and eventually, you see some improvement – because I remember in our first presentation, we were doing badly, but in our second presentation, we realized some improvement, which really encouraged me. It has given me strength to base on for decision making. *DHO Intervention, East*

Data plus knowledge of the network of team actors delineated the decision-making space where the data-informed decisions would have their effect; problem identification showed where team sensitization was needed. Together, they helped managers identify why their standing was either declining or improving and take steps to address the drivers of that change.

### Data and skills improvement addressed the tendency to short-term orientation by presenting the bigger picture and lengthening the outcome terms

The segmentation process often uncovered poor quality data collection, specifically with under-reporting and skills gaps in areas such as budgeting and time-management. Data under-reporting came in the form of the register being ticked for the sake of ticking, without any real understanding of its downstream utility (or even its correspondence to the actual situation), while in control groups, data seemed under-reported because of a lack of funding:

P3: We also have issues of documentation. Some health workers would prescribe INH, but when you go to the IPT register, there is nothing, and that has been letting down our data. We have identified all those gaps; it is not that easy, but we have done so. For example, we have put specific people to ensure that the documentation is done. *DHO Intervention, Southwest*

You see those measures are not consistent because when we have the funds to go and do the contact tracing and follow up, we do it. *HC-IV Control, Southwest*

Participants reported that under-reporting was sometimes also tied to a lack of ownership over the data. Ownership improved with the intervention arm, partly because the data was shared widely with the team and partly because they trained personnel to understand that poor quality data collection compromises service delivery; data collection is more than a mere formality. As a result, this process affected how the teams approached other potential projects. One participant reiterated this point at two separate times during the same FGD:

I think IPT is now one of the indicators of TB that is being tracked … That propels us to engage our frontline health workers that the data must be captured because it is an indicator that shows how we are doing in regard to TB. <*Later*:> We are moving away from the register being ticked for the sake of ticking because you know we are going to introduce another intervention. If the intervention is to be effective, your client must be screened to ensure that he does not have TB disease in case you are to enrol this person for IPT *DHO Intervention, East*

DHOs explained to their teams why data collection is necessary and showed people how it is used; this helped get buy-in and produce robust results, and motivated frontline workers as they prepared for new projects. Beyond engaging the frontline workers, the intervention districts also incorporated regular data-cleaning updates into their team meetings:

We now have monthly review meetings where we even speak about performance in IPT. We have quarterly data cleaning exercises where we also talk about our data. And then of recent, we have introduced at the health centre IV departmental meetings where every Monday of every week, we are supposed to report on our performance where IPT is also inclusive. So, it is integrated into our other reporting opportunities. *DTLS Intervention, East*

Communicating data findings and needs kept everyone involved by highlighting their contributions while soliciting ideas for further improvement. This helped inculcate a culture focused on the long-term vision. It also kept the decision space from growing stagnant due to a posture of mere receptivity and instead encouraged local innovation that could be disseminated more broadly.

In addition to data improvement, intervention teams targeted budgeting and time-management skills to develop:

What I have learnt specifically is to draw a work plan on how to implement IPT whereby we look at what you can do in a specific duration of time. You look at things you can implement either within a quarter or within some few weeks. *DTLS Intervention, East*

For our participants, implementing these skills clarified how decisions get carried out, by whom, and their limits (in terms of time and finances), thereby framing the decision-making space within long-term objectives.

### Self-reliance and empowerment directly promoted movement away from a tendency towards uncertainty avoidance

Empowerment is a feeling that comes from sense of self-efficacy – that one’s actions have a concrete effect in the real world – and moves beyond fulfilled expectations. Initially, the intervention did not match the expectations participants had, as reported in two different FGDs:

I thought that we were going to have activities and there would be a budget and facilitation, there would be fuel. I felt disheartened a bit, but then what is interesting is that for us coming here, we discuss and then agree that this is the action plan, and we go back and do it, and it works. *DHO Intervention Southwest*

For those of us who were in the study and what we are now, at least there is a very big difference that is, the accessibility of our clients has improved, <and> our capacity to use our own data improves every other time we meet for the collaborative meetings because we have the trigger. *DHO Intervention, East*

The evidence before their own eyes about the efficacy of the process, feeling a sense of responsibility for action plans they created, and seeing the effect of using their own data, gave DHOs a sense of empowerment, motivating them to continue despite the initial mismatch between the expected and the experienced. By allowing personnel to use that data to create their own action plans and likewise see the results for themselves, DHOs and DTLSs were able to increase team-wide ownership of the data, tightening workflows:

We also at the same time do data validation when they are bringing their reports and you also look at their reports for IPT and the reporting for other TB activities. And there you let them come up with their action plan on what they should do next specifically for IPT since they are not reporting. *DTLS Intervention, East*

The other thing is the sharing is also evidence-based; it is guided by data. We are talking about the activities that we set out to do. We had a work plan, and there were targets. Given the targets, how much were we able to achieve? – and everybody presents. It is the data now to judge and I find that very informative. *DHO Intervention, Southwest*

By developing a sense of movement within the project through the combination of data ownership and consequent search to identify the root causes of performance issues, teams became more creative at problem-solving, making effective use of resources in the Ugandan healthcare ecosystem as a whole:

We start to ask ourselves questions like; how can we work with IPs? How can we work to ensure that the stocks of INH are there to serve the people, how can we make our health facilities qualified to have the stocks that INH has; so you find that a person uses his/her own resources, the knowledge in terms of the gap and you find that you can add a step and do better. *DHO Intervention, East*

A sense of collective creativity motivated personnel to make the decisions necessary to carry out policy effectively while building their own resources by persuading others, such as Implementation Partners, to join the project. Evidence that data can be used to create one’s own action plans to achieve MoH policies, and the collaboration involved in identifying root problems led to a palpable sense of empowerment among participants. Three respondents in the FGD responded in sequence about the empowerment they felt after the meetings:

P8: To me I can say that it is motivational because you can identify the problem yourself. It motivates you to actually look at the problem yourself and put in place strategies.

P7: And you take the charge, you become responsible, you feel it is yours.

P2: And actually more so to that: you find that your colleagues share best experiences of how they have carried out an activity. There is a way it motivates you to say, ‘I can also do it. If a certain district can perform to this level, then I can also improve.’” *DHO Intervention, Southwest*

Empowerment generates movement and enthusiasm. Decisions are known through their execution, which is defined by movement; buy-in is defined by creative problem-solving in the process of deploying those skills to find partners and identify resources (or resource gaps). For the intervention participants, these skills ultimately rested on the segmentation and root-cause analysis practices becoming habitual practices in the institutional culture.

### Sharing best practices: fostered the collective consensus necessary to alleviate uncertainty avoidance and promote uptake of exemplary models

As P2 noted in the quote above, the prior four practices were consolidated through the requirement that groups present their experiences to colleagues as a way to develop and share best practices. Two participants reported that:

P6: We would share in groups what we decided to do and be able to identify which aspects can lead to progress and see why some interventions did not work and then come up with some solutions. *DHO Intervention, Southwest*

P4: We look at those that have done very well, but there are also strugglers. Then we share those experiences: for those who are doing well, what have we done? Why is it working for you, and <why> is it not working elsewhere? Those that are struggling, what support would you like to get from those that are getting on well? *DHO Intervention, Southwest*

Group sharing gave districts an opportunity to solicit ideas around better practices. Creating a culture of getting buy-in, of listening, and of adapting policies to local contexts primed the presenters to be receptive to other district suggestions. Sharing in groups showed others what decision-making spaces other districts operated within, while putting into place the persuasive skills necessary to increase collective action as local teams.

Participants were universally receptive to learning how they could improve, especially in the context of a friendly rivalry with other districts. As one DTLS noted, ‘ … when you share best practices from the different districts it makes some of us to adopt them and we also use them to succeed in the way we operate’ (*DTLS Intervention, East*).

The overall result of the intervention training, as described by the participants and repeated in many variations, was not only that seeing is believing but also that believing means coming up with your own answers to meet the obstacles you see.

At the beginning, we were not sure whether it was going to work … it works and now we are looking for ways to ensure that it expands to other health facilities and also ensure to enrol more, because those that need to be enrolled are there … We see a lot of gaps in the health centres as we are operating. *DHO Intervention, Southwest*

Seeing action plans succeed gave DHOs and teams a sense of confidence and empowerment, which encouraged them to share these practices. By showing others how their districts solved common problems, they addressed the second root issue, the lack of clarity around the parameters of one’s power to interpret policy implementation in ways which work for the particulars of each district’s unique situation. If one or several other districts were taking a certain approach, that approach could potentially be done or adapted to one‘s own district.

#### Root challenges

*Managers know what to do; but they don’t always know how to do it in culturally appropriate ways.*

The control group informants highlighted two fundamental challenges. The first is an incomplete sense of the range of a DHO’s authority to interpret the implementation of policy (sometimes called ‘decision-making space’). DHOs understood that their role was to implement policy, that this policy came generally from the MoH, and that they were responsible for seeing its uptake in their own districts. However, participants did not have a clear sense of how much they could bring to this role:

The policy is a final document and when it is there, you implement it. So, policy changes are passed from elsewhere and us, we are just implementers, we are not policymakers. *DHO Control, Southwest*

This first issue, therefore, concerned a lack of clarity around when policy implementation shades into policymaking, as when a DHO sets up new protocols to achieve health policy ends.

The second challenge concerned getting buy-in that results in sustained change. While the former challenge is a leadership challenge, this latter is a managerial issue:

The demotivating bit on my side in particular is that regardless of the fact that we go there <to a local health centre> twice for supervision, it is not enough – because the moment you go there <the> second time, you find that some things have not changed. The supervision has to be continuous to make sure that the health workers take on the practices *Health Centre-IV Control, Southwest*

A lack of ownership and buy-in, shown by the need for continuous supervision, seemed to result when mid-level managers were not able to express fully the rationale of a new policy. When health workers understand both the big picture and see improvement in the granular data around retention and cure rates, workers are motivated to sustain the work themselves. This concrete connexion to the patients involved is the final element connecting policy through the person on the ground to the community and was shared by both intervention and control groups. One control DTLS described how this motivates his work, saying,

It’s the passion-the love for what I do, if for instance a patient comes to the facility and misses a service it would haunt me because I would be the one responsible for the welfare of that patient. It is my responsibility to have everything in place, but the scenario of a patient bouncing back makes me feel bad. *DTLS Control, West*

A control DHO also concurred, explaining further that,

Once we have diagnosed the patient we would want to have that patient take the treatment. So the number of patients who finish treatment is very important to me. They shouldn’t only just finish treatment, but they should get cured. So the cure rate is also something that – actually, it is these indicators that really motivate me to ensure that things work … *DHO Control, East*

Yet although this connexion was important to both groups, intervention districts saw sustained change six months after the finish of the national IPT campaign in contrast to the control groups. This suggests that the key practices expressed in the intervention FGDs addressed the root causes at issue by leveraging metric-based decision-making and data on IPT initiation in order to connect those team management elements to the health outcomes of individual patients [21]. The mechanism was succinctly framed by an intervention discussant:

There is a difference between you knowing and it is a completely different thing to implement what you know. So, what this collaboration improved was the implementation and the running and the facilitation of the teams, the sharing of information, the sharing of the burden. … It was those links and intricacies that we discussed in those groups as colleagues *DHO Intervention, Southwest*

## Discussion

The findings from this qualitative analysis of FGD and KII with intervention and control groups scaling up IPT in Uganda move beyond a discussion of goal setting and training objectives to look at the culturally influenced ways participants adopted and applied specific skills to address previously intractable challenges within a decentralized healthcare system. Health system decentralisation has come with mixed results because the complexity of processes involved creates distinct implementation challenges at different levels; generally, decentralisation sees better results at primary and secondary levels rather than tertiary levels like frontline care delivery and disease prevention [28]. This study documents that lack of know-how for implementing skills, and not lack of knowledge *per se*, was the missing link in earlier capacity-building efforts. The common questions participants had coming into the meetings – ‘How do DHOs engage their team?’ ‘Why aren’t the frontline health workers taking up the practices?’ ‘What else can we do?’ and ‘What components are being overlooked?’ – originated first in the lack of clarity surrounding how to implement policy, and second, in the unsettled parameters of district authority by which policy implementation occurs, themes identified in previous studies [29,30].

Intervention sites answered the ‘how’ portion of the above questions by adopting data-based decision-making, which was both motivating and boosted a sense of psychological capability since it caused DHOs to appear more objective as managers and helped them gain team buy-in: they could present data for all to see as a starting point for solving district challenges. This confirms previous findings that wider decision space results from ‘learning by doing’ [31] and works well in a culture of high uncertainty avoidance, in which people are averse to unknown or unpredictable situations [18,32]. Participants were also motivated by the mini-collaboratives, importantly not through individualistic social pressure but through collegial and supportive competitiveness, which functions well in collectivist societies.

District leadership recognized that for their decisions to be sound, the data had to be reliable, and so they implemented processes to clean and validate the data. They also learned to drill down to the specific area where the data showed gaps. Root-cause analysis to pinpoint a problem’s source resulted in teams drawing up workplans, action agendas, and budgets to guide their efforts, all of which seemed to be the missing links in the skill’s know-how. In other words, the mini-collaboratives fostered the skills necessary to recognize capability within the opportunities at hand in a culturally appropriate manner.

Although communication skills are often mentioned as a target skill to improve, our participants discussed not communication so much as empowerment, which *resulted from* improved communication and downward-oriented trust [33,34]. Rather than communication per se, the overlooked element may be the combination of *framing a common task* and then supporting personnel to see it through in a way where everyone can see the results, i.e. by giving people the responsibility of creating and sharing their own pathways to accomplish that task. If capability and opportunity influence the relation between motivation and behaviour [6], this sense of a common task also appealed to cultural values of collective action; agency, in this case, is collective. When district teams created action plans together, implemented them, and saw that they worked, the team was encouraged to trust the manager’s leadership capability. We argue this is also how leadership functions in context, answering a previously open question [1,35] and illustrating the power of local responsiveness to address local challenges [36].

In addition to increased team engagement, sharing best practices allowed districts to evaluate what was going to work in their particular setting, corresponding to findings from other studies [36,37]. DHOs need authority to draw up and test plans – even when they lack financial capacity and limited decision-making space [2]. The mini-collaborations allowed participants to share the process of discovery and receive feedback from other district members, which has also been shown to improve individual skills and consolidate lessons learned from experience [38]. Ultimately, the intervention increased a sense of confidence in meeting the responsibilities of that aim for all participants involved. The result was not only a competitive but also a cooperative enterprise because the participants shared the common aim of improving the health of their districts and the population as a whole. This is a relation between psychological capability and reflexive motivation – motivation derived from planning and evaluation – a relation not clearly expressed previously [5,6].

### Limitations

This paper has three principal limitations. First, we avoided the management/leadership distinction to focus on the practicalities of getting buy-in and teamwork, highlighting practices that fostered psychological capability and reflexive motivation. [33,39] Focusing on the practicalities is an approach taken in diplomacy when ideological problems threaten to forestall movement towards needed, locally impactful solutions while providing a potentially generalizable model for other interventions to replicate. [40]

Second, our data collection sought to evaluate the experience of participants in the intervention, rather than generate evidence for its long-term sustainability. We did not seek to measure empowerment or an increase in self-efficacy; this is an inductive finding based on reports from the DHOs and may reflect a social-desirability bias. Yet a strength of our study is the longitudinal component of seeing the sustainability of IPT in districts which had the intervention in contrast to the rest of the country after a national rollout campaign had ceased.

Third, the difference in data-gathering methods between one-on-one conversations in KIIs and group dynamics in FGDs means that some elements may have been given greater weight in the FGDs to foster discussion and build on what other participants said, while the KIIS in turn may have pursued less commonly mentioned challenges which did not get much ‘air time’ in the FGDs. Both sets of interview and discussion guides were aligned with one another as much as possible, with the exception of questions about how the intervention affected performance, which is the focus of this paper.

### Conclusions and meaning of our results

Promoting sustainable development is a driving motive for decentralisation [41]. Improvement is a complex interplay of factors beyond simple technical capacity. Our participants suggested that capacity building at the mid-manger level must reach further than identifying knowledge gaps to also show people how to implement knowledge they already possess. Although some proponents have advocated for fully systemic changes to address such capacity gaps, evidence shows that changing one block of the system can have effects on the rest of the healthcare system [34]. Generational shifts in top-down vs bottom-up models of healthcare mean structures can lag a generation behind current research; given we are currently in an integrative wave, the district health level is the key place to target as the intersection of MoH directives and oversight of programmes [42]. For these key players, both leadership and management skills are fundamental. Focusing on core practices – rather than competencies – is objectively implementable and measurable at the system level and does not rely on self-assessments of knowledge or pre/post-training skill sets that have been shown to be unreliable [1]. Horizontal accountability, in which mid-level health system managers share their experiences implementing core practices, is one approach that has already made concrete and demonstrably sustainable changes at the district level in Uganda’s decentralized healthcare system.

## Data Availability

Data is available upon request but is not publicly available due to confidentiality concerns.

## References

[1] Johnson O, Begg K, Kelly AH, Sevdalis N. Interventions to strengthen the leadership capabilities of health professionals in Sub-Saharan Africa: a scoping review. Health Policy Plan. 2021;36:117–11. doi: 10.1093/heapol/czaa078 33313871 PMC7938510

[2] Fonn S, Ray S, Couper I, et al. Acceptability and feasibility of inter-related activities to improve agency among African district health managers: a four-country study. Glob Public Health. 2021;17:1–15. doi: 10.1080/17441692.2021.1924220 34097583

[3] Mitchell A, Bossert TJ. Decentralisation, governance and health-system performance: ‘where you stand depends on where you sit’. Devel Policy Rev. 2010;28:669–691. doi: 10.1111/j.1467-7679.2010.00504.x

[4] Mansour W, Aryaija‐Karemani A, Martineau T, et al. Management of human resources for health in health districts in Uganda: a decision space analysis. Int J Health Plann Manage. 2022;37:770–789. doi: 10.1002/hpm.3359 34698403 PMC9298089

[5] Michie S, Van Stralen MM, West R. The behaviour change wheel: a new method for characterising and designing behaviour change interventions. Implementation Sci. 2011;6:1–12. doi: 10.1186/1748-5908-6-42 PMC3096582 21513547

[6] West R, Michie S. A brief introduction to the COM-B model of behaviour and the PRIME theory of motivation. Qeios. 2020. doi: 10.32388/ww04e6.2

[7] Roman TE, Cleary S, McIntyre D. Exploring the functioning of decision space: a review of the available health systems literature. Int J Health Policy Manag. 2017;6:365–376. doi: 10.15171/ijhpm.2017.26 28812832 PMC5505106

[8] Ghuman B, Singh R. Decentralization and delivery of public services in Asia. Policy Soc. 2013;32:7–21. doi: 10.1016/j.polsoc.2013.02.001

[9] Bossert TJ, Beauvais JC. Decentralization of health systems in Ghana, Zambia, Uganda and the Philippines: a comparative analysis of decision space. Health Policy Plan. 2002;17:14–31. doi: 10.1093/heapol/17.1.14 11861583

[10] Massuanganhe J. Economia Política e Política Económica da Governação Descentralizada (Bases Económicas e Fiscais de Sustentabilidade dos Entes Locais). 2022.

[11] Howell-Moroney M. The tiebout hypothesis 50 Years Later: lessons and lingering challenges for metropolitan governance in the 21st Century. Public Adm Rev. 2008;68:97–109. doi: 10.1111/j.1540-6210.2007.00840.x

[12] WHO. Guidelines for intensified tuberculosis case-finding and isoniazid preventive therapy for people living with HIV in resource-constrained settings. Geneva, Switzerland: World Health Organization; 2011.

[13] WHO. Global tuberculosis report 2020. Glob Tuberc Rep: World Health Organization; 2020.

[14] Gupta RK, Lucas SB, Fielding KL, et al. Prevalence of tuberculosis in post-mortem studies of hiv-infected adults and children in resource-limited settings: a systematic review and meta-analysis. AIDS. 2015;29:1987. doi: 10.1097/QAD.0000000000000802 26266773 PMC4568896

[15] Myrtho Casséus R, Gérald Chéry F, Kern A-L. Décentralisation dans les États francophones du Sud. Contexte et mise en œuvre en Haïti. Études caribéennes. 2022;2022. doi: 10.4000/etudescaribeennes.24728

[16] Kristiansen S, Santoso P. Surviving decentralisation?: impacts of regional autonomy on health service provision in Indonesia. Health Policy. 2006;77:247–259. doi: 10.1016/j.healthpol.2005.07.013 16125273

[17] Jabs LB. Collectivism and conflict: conflict response styles in Karamoja, Uganda. Int J Confl Manag. 2005;16(4):354–378.

[18] Rarick C, Winter G, Nickerson I, et al. An investigation of Ugandan cultural values and implications for managerial behavior. Global J Manag Bus Res Adm Manag. 2013;13:1–9.

[19] Crosby R, Noar SM. What is a planning model? An introduction to PRECEDE-PROCEED. J Public Health Dent. 2011;71:S7–S15. doi: 10.1111/j.1752-7325.2011.00235.x 21656942

[20] Onken L. PRECEDE-PROCEED and the NIDA stage model: the value of a conceptual framework for intervention research. J Public Health Dent. 2011;71:S18–S9. doi: 10.1111/j.1752-7325.2011.00221.x 21656945

[21] Kakande E, Christian C, Balzer LB, et al. A mid-level health manager intervention to promote uptake of isoniazid preventive therapy among people with HIV in Uganda: a cluster randomised trial. Lancet HIV. 2022;9:e607–e616. doi: 10.1016/S2352-3018(22)00166-7 35908553 PMC9536151

[22] Johnson-Peretz J, Chamie G, Kakande E, et al. Geographical, social, and political contexts of tuberculosis control and intervention, as reported by mid-level health managers in Uganda: ‘The activity around town’. Soc Sci Med. 2023;338:116363. doi: 10.1016/j.socscimed.2023.116363 37944344 PMC10878310

[23] Christian C, Kakande E, Nahurira V, et al. Mid-level managers’ perspectives on implementing isoniazid preventive therapy for people living with HIV in Ugandan health districts: a qualitative study. BMC Health Serv Res. 2024;24. doi: 10.1186/s12913-024-10803-9 PMC10921742 38454501

[24] Watkins DC. Rapid and rigorous qualitative data analysis: the “RADaR” technique for applied research. Int J Qualitative Method. 2017;16:1609406917712131. doi: 10.1177/1609406917712131

[25] Wilson FP, Dell LD, Anderson GF. Root cause analysis: a tool for total quality management. J Healthc Qual. 1996;18(1):40.

[26] Han E. Harvard business school online. 2023 [2023 Oct 25]. Available from: https://online.hbs.edu/blog/post/root-cause-analysis

[27] Henneberg SC, Mouzas S, Naudé P. Going beyond customers–a business segmentation approach using network pictures to identify network segments. J Bus Market Manage. 2009;3:91–113. doi: 10.1007/s12087-009-0003-y

[28] Suhail A, Gohar A, Steen T. Decentralization reforms in the public health sector in Pakistan. In: Public sector reforms in Pakistan: hierarchies, markets and networks. Cham: Springer International Publishing; 2022. p. 195–222.

[29] Nanyonjo A, Kertho E, Tibenderana J, et al. District health teams’ readiness to institutionalize integrated community case management in the Uganda local health systems: a repeated qualitative study. Global Health Sci Pract. 2020;8:190–204. doi: 10.9745/ghsp-d-19-00318 PMC7326515 32606091

[30] Tabrizi JS, Gholipour K, FarahBakhsh M, et al. Developing management capacity building package to district health manager in northwest of Iran: a sequential mixed method study. J Pak Med Assoc. 2016;66:1385–1391. 27812053

[31] Bossert TJ, Mitchell AD, Janjua MA. Improving health system performance in a decentralized health system: capacity building in Pakistan. Health Syst Reform. 2015;1:276–284. doi: 10.1080/23288604.2015.1056330 31519095

[32] Hofstede G. Dimensionalizing cultures: the Hofstede model in context. Online Readings Phychol Culture. 2011;2:2307–2919.1014. doi: 10.9707/2307-0919.1014

[33] Bradley EH, Taylor LA, Cuellar CJ. Management matters: a leverage point for health systems strengthening in global health. Int J Health Policy Manag. 2015;4:411–415. doi: 10.15171/ijhpm.2015.101 26188805 PMC4493581

[34] Liang Z, Howard PF, Koh LC, et al. Competency requirements for middle and senior managers in community health services. Aust J Prim Health. 2013;19:256–263. doi: 10.1071/PY12041 23007275

[35] Prashanth NS, Marchal B, Devadasan N, et al. Advancing the application of systems thinking in health: a realist evaluation of a capacity building programme for district managers in Tumkur, India. Health Res Policy Syst. 2014;12:42. doi: 10.1186/1478-4505-12-42 25159487 PMC4245764

[36] Drobac PC, Basinga P, Condo J, et al. Comprehensive and integrated district health systems strengthening: the Rwanda population health implementation and training (PHIT) partnership. BMC Health Serv Res. 2013;13:1–13. doi: 10.1186/1472-6963-13-S2-S5 23819573 PMC3668243

[37] Lieberman SS, Capuno JJ, Minh HV. Health decentralization in east asia: some lessons from Indonesia, the Philippines, and Vietnam. UPSE Discussion Papers. 2009 Mar 23.

[38] Klein GA. Sources of power: how people make decisions. Cambridge (MA): MIT press; 2017.

[39] Bolden R, Hawkins B, Gosling J, Taylor S. Exploring leadership: individual, organizational, and societal perspectives. Oxford (UK): Oxford University Press (OUP); 2011.

[40] Ross D. Statecraft: and how to restore America’s standing in the world. New York: Farrar, Straus and Giroux; 2007.

[41] Dansou DH, Carrier M. Décentralisation en Afrique subsaharienne francophone: difficultés des gouvernements, engagement innovant de la société civile. Revue Gouvernance. 2023;20:47–73. doi: 10.7202/1106044ar

[42] Ferreira de Sousa JR, Batista LF, Helal DH. Sobre implementação de políticas públicas: uma revisão sistemática da literatura e agenda de pesquisas. Soc estado. 2022;37:457–487. doi: 10.1590/s0102-6992-202237020004

